# Minutes of the Twenty-Third Annual Meeting of the Mississippi Valley Association of Dental Surgeons

**Published:** 1867-04

**Authors:** 


### MINUTES OF THE TWENTY-THIRD ANNUAL MEETING OF THE MISSISSIPPI VALLEY ASSOCIATION OF DENTAL SURGEONS.

The Association met, pursuant to adjournment, in the lecture room of the Ohio College of Dental Surgery, on Wednesday, the 6th day of March, at 10 o'clock, A. M.

The President, G. W. Keely in the Chair, called the Society to order. On calling the roll, the following members were found to be present, viz:

Drs. A. Berry, J. Taft, H. McCullum, J. P. Ulrey, W. H. Goddard, George Watt, A. S. Talbert, George W. Keely, James Taylor, W. H. Shadoan, C. W. Spaulding, B. D. Wheeler, W. Taft, H. A. Beamer, J. G. Cameron, W. R. Lilly, W. H. Morgan, H. Newington, C. R. Taft, B. F. Rosson, W. G. Redman, W. H. Woodward, C. C. Chittenden, W. W. Hammond, W. C. Duncan, R. A. Mollyneaux, H. R. Smith and M. Wells.

President Keely read an address, giving some details of the history of the Association, and other matters of special interest. The minutes were read, corrected and approved.

The Executive Committee being absent, on motion, the following were appointed by the President to act as an Executive Committee *pro tem.*, viz: Drs. W. H. Morgan, H. A. Smith and H. C. Howells.

An intermission was given for the payment of annual dues.

A resolution was adopted to the effect that the Treasurer notify all members delinquent in the payment of dues for two years of their indebtedness, before erasing their names from the rolls.

On a motion unanimously carried, Dr. W. H. Goddard, of Louisville, Ky., was received into full membership. Dr. Goddard was formerly an active member, but for several years had withdrawn from the professsion.

The Executive Committee reported the following Order of Business:

1. Reports of officers.
2. Election of officers to take place at 11 o'clock, Thursday morning.
3. Miscellaneous business in order at any time.

SUBJECTS FOR DISCUSSION.

1. Mechanical Dentistry. New Inventions.
2. Dental Specialties—their advantages and disadvantages.
3. Operative Dentistry.
4. "What can be accomplished for the arrest of Dental Caries, either by local or general treatment, otherwise than by filling?"
5. Anæsthetics.
6. Papers in order at the opening of each discussion.
7. Reading of an Essay by Professor C. W. Spaulding to be the special order for Thursday evening.

W. H. MORGAN,<br>H. A. SMITH,<br>H. C. HOWELLS, } *Com.*

The Committee to procure the services of a reporter, having engaged one for the full session, their report was received and they discharged.

J. G. CAMERON,<br>W. H. SHADOAN, } *Com.*

Two o'clock, Wednesday afternoon was set apart for the Association to take action in regard to the meeting of the "American Dental Association," to be held in this city in July next.

On motion, the President appointed a Committee to Nominate Candidates for offices for the ensuing year.

Drs. W. H. Morgan, James Taylor and B. F. Rosson were appointed said Committee.

### REPORT ON PRIZE ESSAYS.

The Committee on Prize Essays would respectfully report that but one essay has been presented for their consideration, and that one too late either to comply with the requirements of the Association, or to allow sufficient time to read and examine it.

GEORGE WATT,
H. A. SMITH,
A. S. TALBERT, } *Com.*
G. W. KEELY,
C. M. WRIGHT,

This report was adopted, and the Committee instructed to request the author of the essay presented, to again present it at the next annual meeting.

The same Committee was re-appointed.

The consideration of the adoption of a "Code of Ethics" was made the second special order for the afternoon session.

Adjourned until 2 P. M.

### AFTERNOON SESSION.

President Keely in the Chair. Minutes read and approved.

The Committee on Membership recommended the following names of candidates:

Drs. A. M. Moore, Lafayette, Ind; J. W. Keely, Brookville, Ind; C. R. Butler, Cleveland, Ohio; M. M. Oldham, Springfield, Ohio; D. R. Jennings, Ravenna, Ohio; E. S. Holmes, Grand Rapids, Michigan; J. A. Watling, Ypsilanti, Michigan; S. S. White, Philadelphia, Pa.; D. W. Gill, West Liberty, Ohio; George W. Field, Cincinnati, Ohio; C. M. Kelsey, Mount Vernon, Ohio; J. B. Beauman, Columbus, Ohio; W. C. Stanley, Dublin, Ind.; C. Bradley, Dayton, Ohio; J. Frank McGinnis, Bellefontaine, Ohio; P. T. Clark, La Grange, Texas, all of whom were unanimously elected.

First special order, viz: meeting of the American Dental Association.

On motion of Dr. J. Taft, the Society recommended that no change of time be made, but a change of place in case of the epidemic occurring in the city.

On motion of Dr. Morgan, a committee of three was appointed to raise funds for the purpose of giving a banquet to the American Dental Association. An amendment was offered and accepted, to increase the number to nine, with power to the chairman to make further appointments as may be needed.

Committee appointed: Drs. A. Berry, Cincinnati, Ohio; W. H. Morgan, Nashville, Tenn.; W. G. Redman, Louisville, Ky.; A. W. Moore, Lafayette, Ind.; C. R. Butler, Cleveland, Ohio; A. S. Talbert, Lexington, Ky.; C. H. Harroun, Toledo, Ohio, and C. W. Spaulding, St. Louis, Missouri.

Second special order: adopted the "Code of Ethics" as adopted by the American Dental Association.

First subject for discussion was presented and lightly touched upon.

Adjourned until 11 o'clock, A. M. Thursday.

### MORNING SESSION, SECOND DAY.

Adjourned until 2 o'clock, P. M.

### AFTERNOON SESSION.

Met pursuant to adjournment, President Keely in the Chair. Minutes read and approved.

First subject resumed—"Mechanical Dentistry."

On motion, the discussion was suspended, and the Association went into an election of officers, which resulted as follows:

*President*—Dr. W. H. Goddard, Louisville, Ky.

*Vice President*—Dr. J. A. Watling, Ypsilanti, Michigan.

*Recording Secretary*—Dr. George W. Field, Cincinnati, O.

*Corresponding Secretary*—C. W. Wright, Cincinnati, O.

*Treasurer*—B. D. Wheeler, Cincinnati, O.

Dr. Morgan was appointed to conduct the President elect to the Chair.

Dr. Goddard not being present, Dr. Keely was requested to act *pro tem.*

On motion, the report of the Nominating Committee was referred back, with instructions to nominate standing committees.

President Keely, on retiring, introduced the President elect with a few remarks highly complimentary to the latter, which were fully endorsed by the Association. President Goddard replied by a well-timed expression of thanks to the Association.

The Committee on Inventions were requested to present for exhibition the articles in their possession.

ARTICLES EXHIBITED.

1. Several sets of teeth, plain and gum, of recent improved molds, prepared by Dr. S. S. White, for the Paris Exposition.

2. Rubber burs and scrapers, for lathe and hand.

3. An automatic mallet, with such an arrangement of springs that the fall of the hammer produces an elastic stroke, like that of the hand mallet. Dr. W. G. Redman, of Louisville, Ky., is the inventor. This was esteemed to be an improvement over all others heretofore exhibited.

4. A benzine gas generator, by J. H. Hall.

5. A spray producer, by the same.

The former was much admired.

6. A combined lip and cheek holder and duct compressor, by Dr. C. Bradley, Dayton, Ohio. This appliance was looked upon as the *ne plus ultra* of "Dentist's Assistants."

On motion of Dr. Talbert, a vote of thanks was extended to Dr. Bradley for his valuable invention. Dr. C. W. Spaulding expressed his appreciation of this appliance in the highest terms. In this he was heartily endorsed by Drs. Watt, Keely, Talbert and others.

8. A "piston plugger," exhibited by Dr. Keely on behalf of Dr. Poore, of Dubuque, Iowa. This instrument not being in good order, its merits were not fully developed.

On motion of Dr. Will Taft, the President appointed a committee of two to nominate delegates to the American Dental Association. The Chair appointed Drs. W. H. Morgan and Will Taft.

The second subject for discussion was announced as in order: "Dental Specialties—their advantages and disadvantages."

Adjourned to 7½ o'clock, P. M.

EVENING SESSION, 7½ O'CLOCK.

President Goddard in the Chair. Minutes read, corrected and approved.

Second subject resumed and discussed briefly.

Third subject taken up : "Operative Dentistry."

Dr. Talbert asked the question: "When the first permanent molar is found so badly decayed as to be past preservation, previous to the eruption of the second molar, is its immediate extraction advisable?"

Professor C. W. Spaulding read an essay on "The Undulatory Theory of the Nerve Forces."

On motion of Dr. Talbert, a vote of thanks was extended to Dr. Spaulding, and a copy requested for publication.

This essay called forth an animated discussion on the nerve force; also, on the action of the sun's rays as essential to the preservation of health, Drs. Taft, Spaulding, Watt and S. S. White being much interested.

Discussion closed, and the Association adjourned until 9 o'clock, A. M., Friday.

THIRD DAY, MORNING SESSION.

President in the Chair. Minutes read and approved.

Third subject resumed and discussed to considerable length.

Dr. Spaulding disparaged the use of cotton for filling apex of fang, as the chemical action of the creosote formed the carbolate of albumen in a few moments as perfectly as it could in months.

The rules were suspended for the introduction of miscellaneous business.

The Nominating Committee reported as follows:

*Executive Committee.*—Drs. G. W. Keely, W. G. Redman and D. R. Jennings.

*Committee on Membership.*—Drs. A. Berry, G. Watt and A. M. Moore.

*Publishing Committee.*—Drs. J. Taft, N. W. Williams and C. M. Kelsey.

W. H. MORGAN, } *Com.*

WILL TAFT, }

The report accepted and adopted.

The following were appointed delegates to the American Dental Association: Drs. R. A. Mollyneaux, H. McCullum, George W. Field, J. P. Ulrey, M. Decamp, W. A. Pease, Joseph Richardson, J. F. Johnson, N. W. Williams, R. Corson, W. R. Lilly, F. C. Eddleman, B. F. Rosson, C. C. Chittenden, A. M. Moore, D. R. Jennings, Francis Peabody, H. Newington, C. Bradley, W. H. Woodward, D. W. Gill, J. C. Ross, W. H. Morgan, R. E. Taylor, C. N. Woodward, G. N. Priest, J. Keely, D. Phillips and W. C. Stanley.

Dr. Francis Peabody, of Louisville, was presented as a candidate for membership, and elected.

A motion to pass to the consideration of the rubber question was carried.

The general expression of feeling indicated a determination to contest the claims of the Rubber Company.

The Treasurer was authorized to pay Dr. Will Taft the sum of twenty dollars as compensation for copying the minutes.

The Committee on Revising the Membership Roll was continued; Drs. Will Taft and C. M. Wright, Committee.

The amendments proposed at the last annual meeting were acted upon, and declared adopted.

A motion to remunerate the janitor for services rendered was carried.

The following proposition was presented by Drs. W. H. Morgan, of Nashville, Tenn., and S. S. White, of Philadelphia, Pa.:

We, the undersigned, offer a prize of two hundred dollars ($200), for the most approved essay on "THE BEST MEANS OF PRESERVING AND PROMOTING THE HEALTH OF DENTISTS."

Essays competing for this prize are to be referred to a Special Committee of five, to be appointed by this Association. They must be in the hands of the Chairman of said Committee as early as January 1, 1868.

This Committee is to be governed by the same rules as that appointed on General Prize Essays.

It is expected that the essay receiving the prize will consider the diseases to which Dentists are especially liable; their causes, modifying circumstances, prevention, etc., etc., including the subjects of ventilation, location of office, operating chairs, &c.

W. H. MORGAN,
SAMUEL S. WHITE.

The Special Prize Essay Committee shall consist of the following: Drs. George Watt, C. W. Spaulding, G. W. Keely, W. G. Redman and S. Driggs.

Drs. Taft and Berry were appointed a Committee on Reporters for next annual meeting.

The following resolution was adopted:

*Resolved*, That it shall be the duty of the Recording Secretary to notify all committees and essayists of their appointment at least two months before each annual meeting.

The following amendment to the By-Laws was proposed, March 7, 1867:

"It shall be the duty of the Recording Secretary to notify members of committees, essayists and delegates of their appointment within one month after the meeting at which they were appointed.

Adjourned to meet in March, 1868.

GEORGE W. FIELD, *Recording Secretary.*

---

